# C-C motif chemokine ligand 2 promotes myogenesis of myoblasts via the AKT-mTOR pathway

**DOI:** 10.18632/aging.204451

**Published:** 2022-12-27

**Authors:** Mi Kyung Kwak, Eun Suk Ha, Jiwoo Lee, Yun Mi Choi, Beom-Jun Kim, Eun-Gyoung Hong

**Affiliations:** 1 Division of Endocrinology and Metabolism, Department of Internal Medicine, Hallym University Dongtan Sacred Heart Hospital, Hwaseong-Si, Gyeonggi-Do 18450, Korea; 2 Division of Endocrinology and Metabolism, Asan Medical Center, University of Ulsan College of Medicine, Songpa-Gu, Seoul 05505, Korea

**Keywords:** CCL2, CCR2, myogenesis, low muscle mass, aging

## Abstract

Muscle mass decreases with aging, while the C-C motif chemokine ligand 2 (CCL2) increases with aging; in this context, CCL2 can be considered a potential aging-promoting factor. Thus, CCL2 knockout mice are expected to exhibit anti-aging effects including protection against loss of muscle mass. However, instead, muscle amount and recovery of damaged muscles are decreased in CCL2 knockout mice. Therefore, we hypothesized that increasing CCL2 in the elderly might be related to compensation for loss of muscle mass. To confirm the relationship between muscle and CCL2, we sought to establish the role of CCL2 in C2C12 cells and Human Skeletal Muscle Myoblast (HSMM) cells. The myotube (MT) fusion index increased with CCL2 compared to 5day CCL2 vehicle only (27.0 % increase, P<0.05) in immunocytochemistry staining (ICC) data. CCL2 also restored MTs atrophy caused by dexamethasone (21.8 % increase, P<0.0001). p-mTOR/mTOR and p-AKT/total AKT increased with CCL2 compared to CCL2 vehicle only (18.3 and 30.5% increase respectively, P<0.05) and decreased with CCR2-siRNA compared to CCL2 (38.9 % (P<0.05) and 56.7% (P<0.005) reduction respectively). In conclusion, CCL2 positively affects myogenesis by CCR2 via AKT-mTOR signaling pathways. CCL2 might have potential as a therapeutic target for low muscle mass and muscle recovery.

## INTRODUCTION

Aging leads to deterioration in various organs, and this phenomenon gradually worsens over time [1]. Aging is also a major cause of low muscle mass and/or sarcopenia [2]. In sarcopenia, the amount of skeletal muscle and strength decrease [3]. When aging or aging-related diseases occur, quality of life decreases due to lost muscle strength, general weakness, and limited activity [4]. However, no drugs have been approved for the treatment of low muscle mass and/or sarcopenia. Therefore, many researchers have sought to identify factors that affect muscle and establish their functions, but more research is needed to better address age-related muscle waste and/or sarcopenia.

Studies within the framework of the heterochronic parabiosis model provide critical insight into aging [5, 6] by making it possible to assess factors that increase respectively in two individuals under different conditions. It is also possible to check the change in the biological level of various factors measured *in vivo* or the change in the phenotype of the individual, after two objects have been connected.

Therefore, studies using this model reveal the importance of factors that circulate in plasma [5, 6]. A study to verify the existence and role of factors related to aging have confirmed factors that are increased on proteome analysis of plasma from elderly and parabiosis mice, and one of these factors was C-C motif chemokine ligand 2 (CCL2) [5]. CCL2 is a C-C motif cytokine, also called MCP-1 that is mainly associated with immune regulation and the inflammatory response [7]. CCL2 exhibits chemotactic activity for monocytes and basophils [8, 9]. It has been implicated in the pathogenesis of diseases characterized by monocytic infiltrates [10], such as psoriasis, rheumatoid arthritis, and atherosclerosis [11–13].

In this context, CCL2 can also be considered a potential aging-promoting factor. CCL2 knockout mice are expected to show anti-aging effects including protection against loss of muscle mass. However, interestingly, one study reported that CCL2 promoted recovery of damaged muscle by recruiting monocytes following acute muscle injury. That report showed that the muscle volume of CCL2 knockout mice decreased, and the area of damaged muscle recovery was also reduced [14, 15]. Another study confirmed that eight genetic mutations in the *CCL2* and *CCR2* genes were related to skeletal muscle strength and muscle response to resistance training [16].

Because CCL2 increases with aging [15, 17], controversy remains about its action on muscles. Furthermore, despite the possible role of CCL2 in muscle metabolism, the effects of CCL2 on skeletal muscle cells and the cellular pathways involved in those processes remain unexplored. Therefore, we hypothesized that increasing CCL2 in the elderly or during muscle injury might be related to compensation for loss of muscle mass. We assumed that CCL2 promotes muscle healing. We investigated the roles of CCL2 in the C2C12 cell line and Human Skeletal Muscle Myoblast (HSMM) cells, as representative skeletal muscle cells, to clarify that possibility.

## RESULTS

### C-C motif chemokine ligand 2 (CCL2) affects cell viability, proliferation, migration, and myogenesis in mouse C2C12 myoblast (MB) and differentiation in myotube (MT)

**Figure 1:**
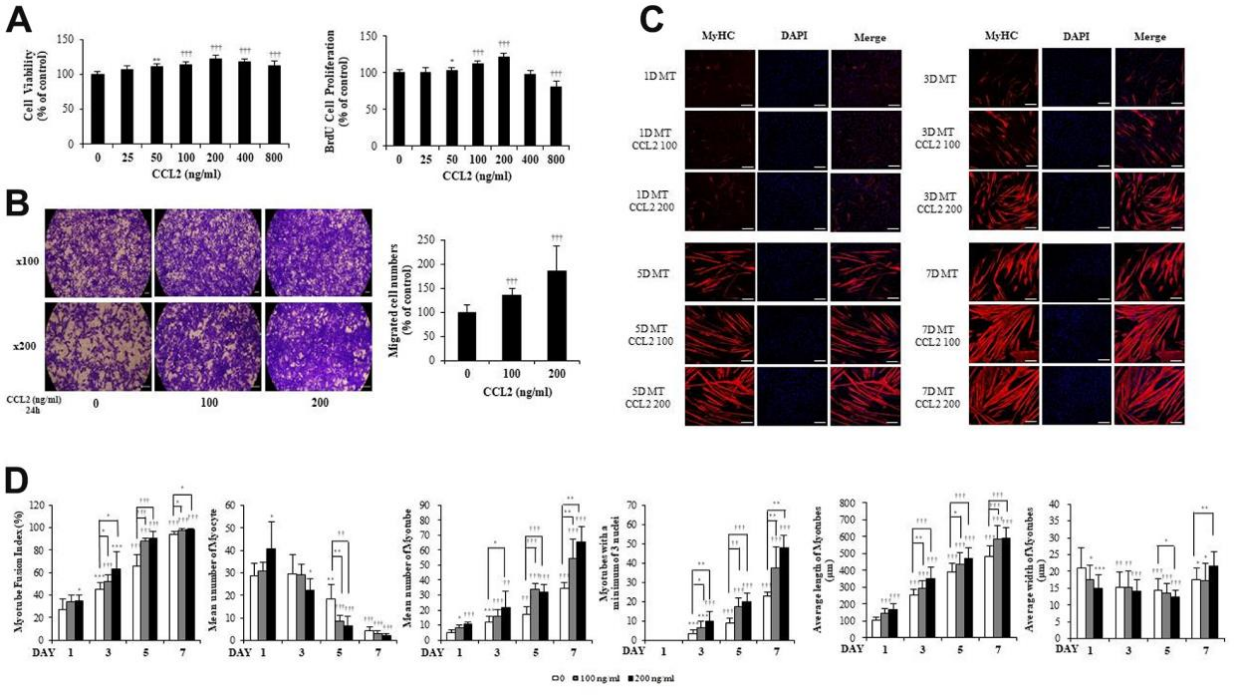
**C-C motif chemokine ligand 2 (CCL2) promotes cell viability, proliferation, migration, and myogenesis in mouse C2C12 myoblasts (MBs).** (**A**) MBs viability on Cell Counting Kit-8 (CCK-8) assay and MBs proliferation on 5-bromo-2′-deoxy-uridine (BrdU) assay. (**B**) MBs migration on Transwell migration assay. (**C**) MBs were differentiated into myotubes (MTs) with 2% horse serum after exposure to the indicated concentrations of recombinant CCL2 for the specified number of days. MTs were stained with anti-myosin heavy chain (MyHC) antibody, and the nuclei were counterstained with 4, 6-diamidino-2-phenyindole (DAPI). Scale bars, 100 μm. (**D**) Quantitative results per field are presented. Number of experiments (No.) of (**A**, **D**) = 4 times, respectively. No. of (**B**, **C**) = 6 times, respectively. Data are expressed as mean ± standard deviation (SD). (**A**, **B**) *P<0.05, **P<0.005, ***P<0.0005, †P<0.01, ††P<0.001, †††P<0.0001 vs. CCL2 '0' (Vehicle: PBS + 0.1% BSA). (**C**, **D**) *P<0.05, **P<0.005, ***P<0.0005, †P<0.01, ††P<0.001, †††P<0.0001 vs. Day 1 of CCL2 '0' (vehicle: PBS + 0.1% BSA), or vs. same day data (represented with lines).

MBs viability was determined using the Cell Counting Kit-8 (CCK-8) assay (DOJINDO, Kumamoto, Japan). And MBs proliferation was confirmed with the 5-bromo-2′-deoxy-uridine (BrdU) assay (Roche, Germany), relative expression of PCNA (NM_011045.2), and Cyclin D1 (BC044841.1). Within the maximum CCL2 concentration of 800 ng/ml, the greatest increase in viability and proliferation was observed at 200 ng/ml of CCL2 compared with CCL2 vehicle only (21.4 % increase, P<0.0001) (Figure 1A). The relative expression of PCNA and Cyclin D1 increased in a concentration-dependent manner in the CCL2 treatment group compared to the CCL2 vehicle only, and the values were the highest at 200 ng/ml of CCL2 (20.6 % increase, P<0.0001 and 28.0% increase, P<0.001, respectively) (Supplementary Figure 1). Using the concentrations mentioned in other papers [14, 15], 0, 100, and 200 ng/ml were selected based on our CCK-8 and BrdU assay experiments. To investigate cell migration capacity, we conducted a Boyden chamber migration assay. CCL2 increased the migration capacity of MBs compared with CCL2 vehicle only (84.9 % increase, P<0.0005) (Figure 1B).

MBs were differentiated into MTs in 2% horse serum after exposure to the indicated concentrations of recombinant CCL2 each day. MTs were stained with anti-myosin heavy chain (MyHC) antibody, and the nuclei were counterstained with 4,6-diamidino-2-phenyindole (DAPI). MTs lengthened as CCL2 concentration increased and differentiation time was extended, allowing us to surmise that MTs differentiation was progressing (Figure 1C).

Next, we examined MTs differentiation parameters with CCL2 in immunocytochemistry staining (ICC) data. We set 640 x 540 um^2^ as one field and analyzed 6 to 8 fields for each condition. If there was one nucleus, a cell was considered a myocyte, and two nuclei, a myotube. The fusion index increased with CCL2 exposure compared to 5day CCL2 vehicle only (27.0 % increase, P<0.05). MTs length also increased significantly compared to 5day CCL2 vehicle only (13.1 % increase, P<0.0001) from day 1 to day 7. The number of MTs and the number of mature MTs with three or more nuclei increased in the same pattern. Also, as MTs fusion progressed from day 1 to day 7 of differentiation, the number of myocytes gradually decreased. MTs elongation increased significantly, but MTs width did not show a distinct difference through day 5 (Figure 1D). Collectively, these data indicate that CCL2 contributes to myogenesis by stimulating viability, proliferation, migration, and differentiation.

### CCL2 affects cell viability, proliferation, and myogenesis in primary human cells, HSMM (human skeletal muscle myoblast) and differentiation in MT

**Figure 2:**
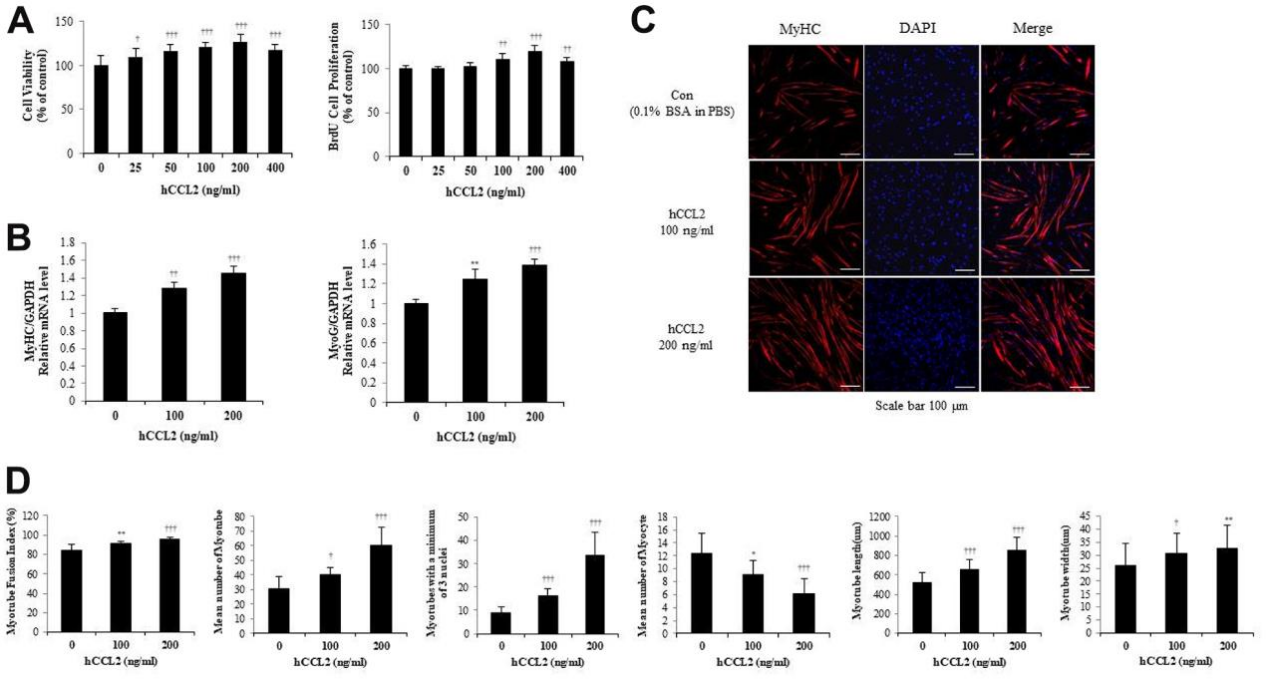
**Recombinant human C-C motif chemokine ligand 2 (hCCL2) promotes cell viability, proliferation, and myogenesis in human skeletal muscle myoblast (HSMM) primary human cells.** (**A**) HSMM cell viability on Cell Counting Kit-8 (CCK-8) assay and proliferation on 5-bromo-2′-deoxy-uridine (BrdU) assay. (**B**) Quantitative reverse-transcription polymerase chain reaction analyses (qRT-PCR) of myogenin and myosin heavy chain (MyHC) as a marker for myotube (MT) formation. (**C**) MTs were stained with an anti-MyHC antibody, and the nuclei were counterstained with 4, 6-diamidino-2-phenyindole (DAPI). Scale bars, 100 μm. (**D**) Quantitative results per field are presented. The number of experiments (No.) of (**A**, **B**) = 4 times, respectively. No. of (**C**, **D**) = 6 times, respectively. Data are expressed as mean ± standard deviation (SD). (**A**, **B**) *P<0.05, **P<0.005, ***P<0.0005, †P<0.01, ††P<0.001, †††P<0.0001 vs. hCCL2 '0' (Vehicle: PBS + 0.1% BSA). (**C**, **D**) *P<0.05, **P<0.005, ***P<0.0005, †P<0.01, ††P<0.001, †††P<0.0001 vs. Day 5 of hCCL2 '0' (vehicle: PBS + 0.1% BSA).

To validate the mouse cell line studies, HSMM viability was determined using the CCK-8 assay, and HSMM proliferation was confirmed with the BrdU assay, as with the experiments with C2C12. Within the maximum recombinant human CCL2 (hCCL2) concentration of 400 ng/ml, the most significant increase in viability and proliferation was observed at 200 ng/ml of hCCL2 compared with hCCL2 vehicle only (26.7 % increase, P<0.0001) (Figure 2A).

To investigate how hCCL2 affects the RNA expression of MTs markers, hCCL2 and vehicle (phosphate-buffered saline [PBS] and 0.1% bovine serum albumin solution [BSA]) were administered to MTs. HSMM were differentiated into MTs in 2% horse serum after exposure to the indicated concentrations of hCCL2 each day. Quantitative reverse-transcription polymerase chain reaction analyses (qRT-PCR) at five days showed that myogenin increased dependent on hCCL2 concentration (39.0 % increase, P<0.0001). MyHC increased hCCL2 concentration-dependent manner compared to a 5day CCL2 vehicle only (45.7 % increase, P<0.0001) (Figure 2B).

Next, MTs were stained with MyHC antibody, and the nuclei were counterstained with DAPI. MTs lengthened as hCCL2 concentration increased, allowing us to presume that MTs differentiation was progressing (Figure 2C). We examined MTs differentiation parameters with hCCL2 in ICC data. The fusion index increased with hCCL2 exposure compared to hCCL2 vehicle only (14.2 % increase, P<0.0001). MTs length increased significantly compared to hCCL2 vehicle only (65.3 % increase, P<0.0001). The number of MTs and the number of mature MTs with three or more nuclei increased in the same pattern (97.7 % increase, P<0.0001 and 277.5% increase, P<0.0001, respectively). As MTs fusion progressed, the number of myocytes gradually decreased (49.2 % decrease, P<0.0001). Also, MTs width increased significantly (6.9 % decrease, P<0.005) (Figure 2D). These data indicate that hCCL2 contributes to myogenesis by stimulating viability, proliferation, and differentiation.

### Transcription factor expression during myogenesis with CCL2

**Figure 3:**
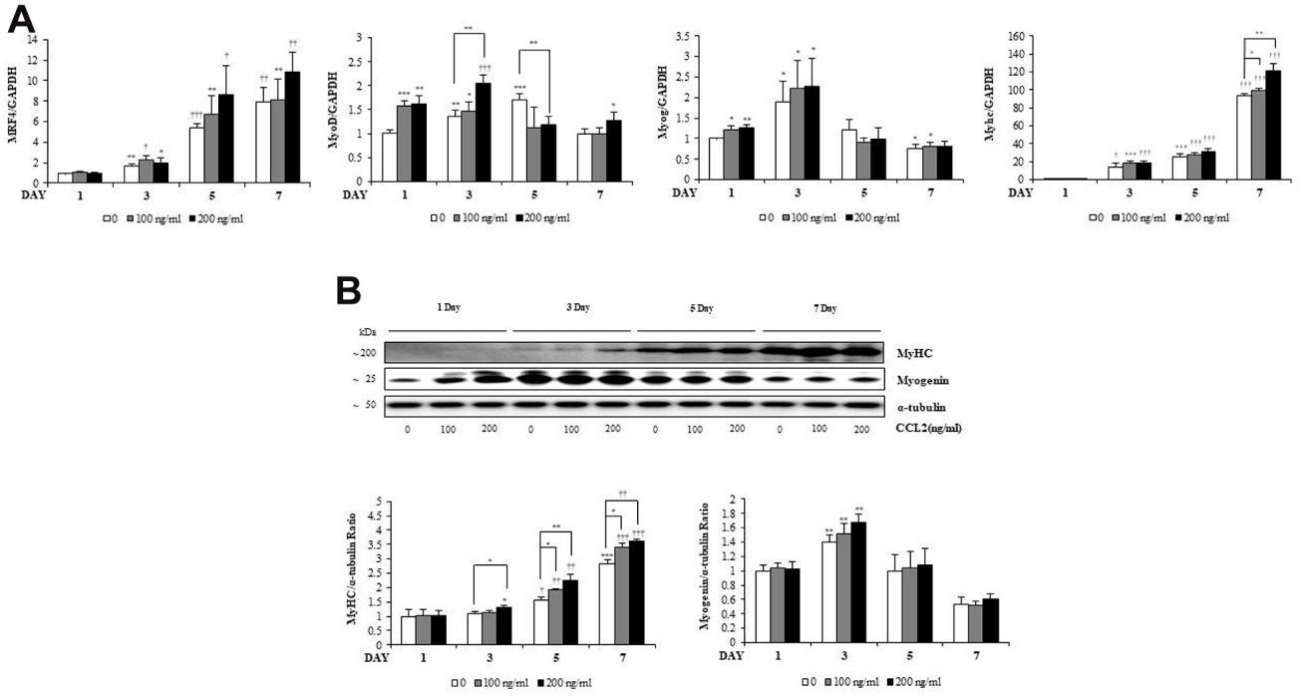
**C-C motif chemokine ligand 2 (CCL2) effects on transcription factors during myogenesis.** (**A**) Quantitative reverse-transcription polymerase chain reaction analyses (qRT-PCR) of MRF4, MyoD, myogenin, and myosin heavy chain (MyHC) as a marker for myotube (MT) formation. (**B**) Western blot of MyHC and myogenin in C2C12 cells with 2% horse serum in the presence of the indicated concentrations of recombinant CCL2 for the specified number of days. Number of experiments of (**A**, **B**) = 4 times, respectively. Data are expressed as mean ± standard deviation (SD). (**A**, **B**) *P<0.05, **P<0.005, ***P<0.0005, †P<0.01, ††P<0.001, †††P<0.0001 vs. day 1 of CCL2 '0' (vehicle: PBS + 0.1% BSA), or vs. same day data (represented with lines).

To investigate how CCL2 affects the RNA expression of MTs markers, qRT-PCR was performed. MRF4 tended to increase, dependent on time and CCL2 concentration (Figure 3A). MyoD increased for three days, until the MTs matured, and then decreased after that. Myogenin increased for three days and decreased after five days. These findings can be visualized because the number of committed myocytes also decreased from day 5. MyHC, a late-stage marker of fusion that reflects the change from committed myocytes to mature MTs, increased in a differentiation-day- and CCL2 concentration–dependent manner compared to 7day CCL2 vehicle only (22.8 % increase, P<0.005) (Figure 3A). Western blot analyses of myogenin and MyHC show that same pattern (Figure 3B). To determine protein synthesis activity during myogenesis after CCL2 treatment, the SUnSET assay was performed. Western blot analysis to quantify relative puromycin incorporation by CCL2 showed that CCL2 increased protein synthesis during myogenesis compared to 3day CCL2 vehicle only (13.4 % increase, P<0.05) (Supplementary Figure 2). All data regarding the RNA expression of transcription factors during MTs differentiation indicated that CCL2 enhances MTs formation, differentiation, and fusion.

### CCL2 receptor in muscle cells

**Figure 4:**
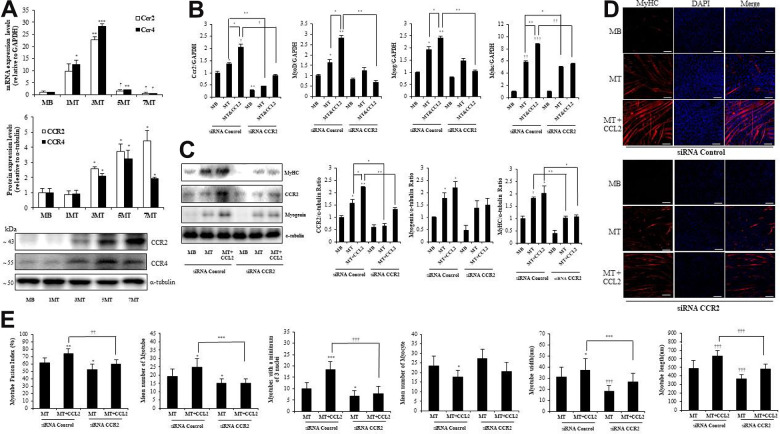
**C-C chemokine receptor 2 (CCR2) is the major receptor for C-C motif chemokine ligand 2 (CCL2) in muscle cells.** Mouse C2C12 myoblasts (MBs) were differentiated into myotubes (MTs) with 2% horse serum for the indicated number of days. (**A**) Quantitative reverse-transcription polymerase chain reaction (qRT-PCR) and western blot analyses were performed to determine the expression of CCR2 in C2C12 cells cultured with 2% horse serum for the specified number of days. (**B**) Mouse C2C12 MBs were differentiated into MTs with 2% horse serum in the presence or absence of recombinant CCL2 and/or CCR2 inhibitor for three days. qRT-PCR results for CCR2, MyoD, MyoG, and myosin heavy chain (MyHC) in C2C12 cells. (**C**) Western blots of CCR2, MyHC, and myogenin in C2C12 cells cultured with 2% horse serum in the presence or absence of recombinant CCL2 and/or CCR2 inhibitor for three days. (**D**) MTs were stained with anti-MyHC antibody, and the nuclei were counterstained with 4, 6-diamidino-2-phenyindole (DAPI). Scale bars, 100 μm. (**E**) Quantitative results per field are presented. Number of experiments (No.) of (**A**–**C**) = 4 times, respectively. No. of (**D**, **E**) = 6 times, respectively. Data are expressed as mean ± standard deviation (SD). (**A**) *P<0.05, **P<0.005, ***P<0.0005, †P<0.01, ††P<0.001, †††P<0.0001 vs. MB. (**B**, **C**) *P<0.05, **P<0.005, ***P<0.0005, †P<0.01, ††P<0.001, †††P<0.0001 vs. siRNA Control MB, or vs. siRNA Control MT or siRNA Control MT & CCL2 (represented with lines). (**E**) *P<0.05, **P<0.005, ***P<0.0005, †P<0.01, ††P<0.001, †††P<0.0001 vs. siRNA Control MT, or vs. siRNA Control MT & CCL2 (represented with lines).

Among the potential receptors that could mediate the action of CCL2 [18], CC chemokine receptor 2 (CCR2) (not CCR4) was strongly expressed in both MBs and MTs (Figure 4A). In line with those results, CCL2 interacted directly with CCR2 in muscle cells. qRT-PCR indicated that CCR2-siRNA attenuated recombinant CCL2–induced myogenesis compared to siRNA control MTs & CCL2 (42.7 % reduction, P<0.001) (Figure 4B). Western blot analyses of transcription factor expression showed that same pattern compared to siRNA control MTs & CCL2 (47.0 % reduction, P<0.05) (Figure 4C). MTs differentiation parameters in the ICC data revealed that CCR2-siRNA decreased MTs differentiation (Figure 4D). Quantitative results per field also indicated that the presence of CCR2-siRNA attenuated CCL2-induced effects (Figure 4E). These findings suggest that CCR2 mediates the effects of CCL2 on myogenic differentiation. The CCL2 concentration used here was 200 ng/ml.

### CCL2 effects on MTs damaged by dexamethasone

**Figure 5:**
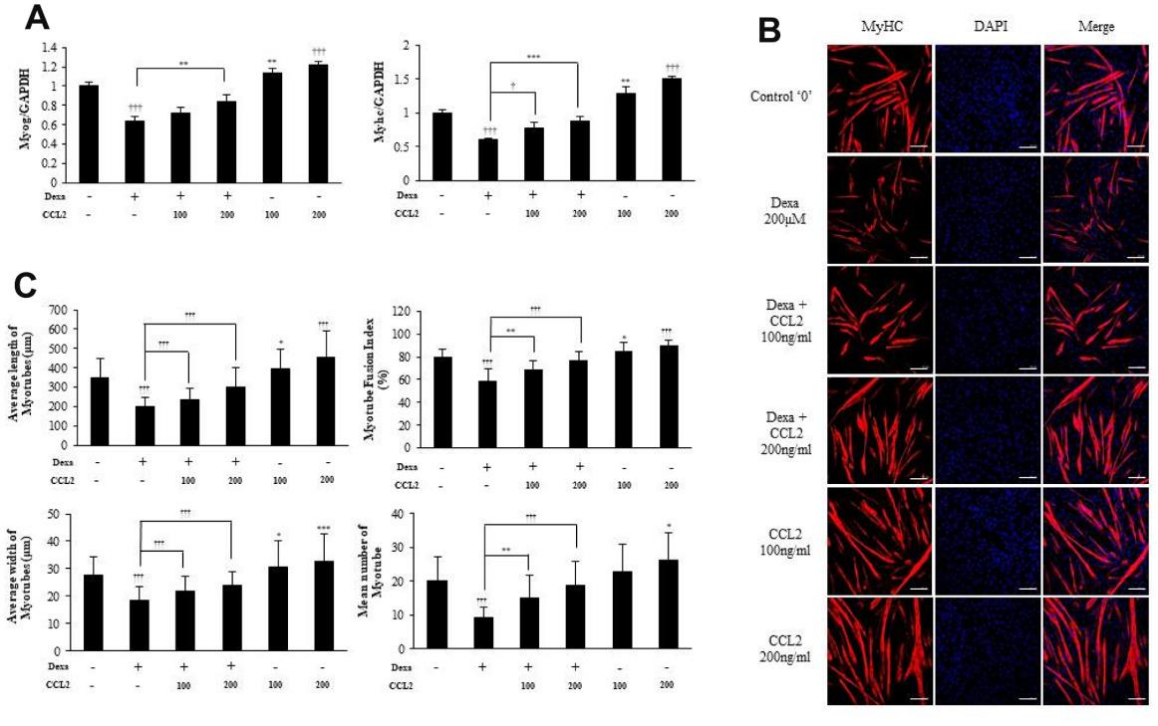
**C-C motif chemokine ligand 2 (CCL2) restores myotubes (MTs) damaged by dexamethasone.** (**A**) Quantitative reverse-transcription polymerase chain reaction analyses (qRT-PCR) of myogenin and myosin heavy chain (MyHC) in C2C12 cells treated with 2% horse serum, with and without MTs damaged by dexamethasone or the indicated concentrations of recombinant CCL2 for 2 days. (**B**) MTs were stained with anti-MyHC antibody, and the nuclei were counterstained with 4,6-diamidino-2-phenyindole (DAPI). Scale bars, 100 μm. (**C**) Quantitative results per field are presented. Number of experiments (No.) of (**A**) = 4 times. No. of (**B**, **C**) = 6 times, respectively. Data are expressed as mean ± standard deviation (SD). (**A**, **C**) *P<0.05, **P<0.005, ***P<0.0005, †P<0.01, ††P<0.001, †††P<0.0001 vs. CCL2 '–' & Dexa ‘– ‘(vehicle: PBS + 0.1% BSA), or vs. CCL2 '–' & Dexa 200μM (represented with lines).

To confirm the importance of CCL2 in protection against myotube atrophy, we induced MTs atrophy with dexamethasone [19, 20] which impairs RNA expression of the transcription factors needed for MTs differentiation. We found that CCL2 restored the damage to MTs caused by dexamethasone (21.8 % increase, P<0.0001) (Figure 5A). The ICC data also show that MTs differentiation was impaired by dexamethasone, and that CCL2 restored myotube atrophy (Figure 5B, 5C). Treating MTs with CCL2 significantly reversed dexamethasone-mediated MTs atrophy.

### Identification of the signal-mediating role of CCL2 during myogenesis

**Figure 6:**
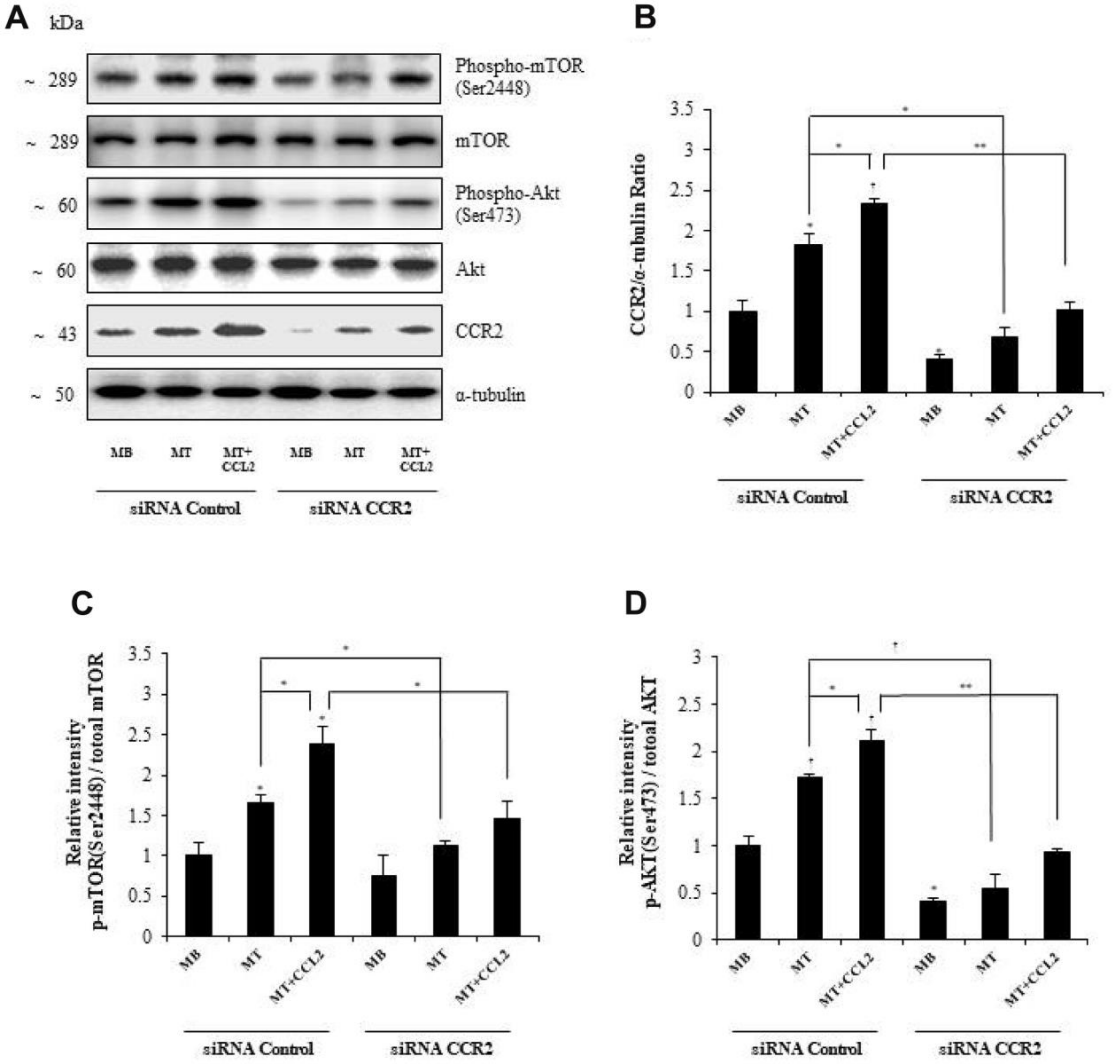
**Effects of C-C motif chemokine ligand 2 (CCL2) on myogenesis are mediated by AKT-mTOR signal stimulation.** (**A**, **B**) Western blot analyses to determine the activity of signals related to myogenesis in C2C12 cells after treatment with 200 ng/ml CCL2. (**C**, **D**) Quantitative reverse-transcription polymerase chain reaction analyses (qRT-PCR) of the relative intensity of *p*-mTOR/mTOR or *p*-AKT/total AKT in C2C12 cells treated with 2% horse serum in the presence or absence of recombinant CCL2 and/or CCR2 inhibitor for three days. Number of experiments (No.) of (**A**–**D**) = 4 times, respectively. Data are expressed as mean ± standard deviation (SD). (**B**–**D**) *P<0.05, **P<0.005, ***P<0.0005, †P<0.01, ††P<0.001, †††P<0.0001 vs. siRNA Control MB, or vs. siRNA control MT, or vs. siRNA control MT & CCL2 (represented with lines).

To determine the mechanism by which CCL2 affects myogenesis, we focused on the AKT-mTOR signaling pathways, which are related to protein synthesis in myogenesis [21]. Western blot analyses revealed that CCR2 expression increased with CCL2 administration and decreased with CCR2-siRNA exposure (Figure 6A, 6B, respectively). The activity of p-mTOR/mTOR and p-AKT/total AKT also increased with CCL2 compared to CCL2 vehicle only (18.3 and 30.5% increase respectively, P<0.05) and decreased with CCR2-siRNA compared to CCL2 (38.9 % (P<0.05) and 56.7% (P<0.005) reduction respectively) (Figure 6C, 6D, respectively). These results indicate that AKT-mTOR is a crucial signal regulating the effects of CCL2 on myogenesis. To detect whether the effects of CCL2 promoting myogenesis can be blocked by the inhibitor, we conducted Western blot analyses with AKT-mTOR inhibitor (Supplementary Figure 3A). The activity of p-mTOR/mTOR and p-AKT/total AKT decreased with Akt inhibitor compared to CCL2 (56.2% (P<0.005) and 94.3% (P<0.005) reduction respectively) (Supplementary Figure 3B). And also, the relative intensity of MyHC and myogenin decreased with Akt inhibitor compared to CCL2 (60.5% (P<0.05) and 67.8% (P<0.01) reduction, respectively) (Supplementary Figure 3C).

## DISCUSSION

Because previous studies showed a possibility of the relationship between skeletal muscle and CCL2, we examined the effect of CCL2 on myogenesis. We found that CCL2 promotes the viability, proliferation, migration, and myogenesis of myoblasts and promotes differentiation of myotubes. And these effects of CCL2 on myogenesis occur through the CCR2, and the AKT-mTOR signaling pathways are the main pathways. To the best of our knowledge, this is the first study to provide evidence that CCL2 can have beneficial effects on myogenesis in the C2C12 and HSMM cell line. Our findings suggest that CCL2 might help prevent and reverse muscle mass loss associated with aging.

C-C motif cytokine CCL2 and CC chemokine receptor CCR2 are expressed on endothelial cells; *in vitro*, CCL2 production increases after injury [22]. CCL2 and CCR2 regulate immune cell recruitment and function during muscle regeneration [7, 14, 23]. Previous studies have shown a clear association between the inflammatory response and subsequent repair processes in skeletal muscle [24, 25]. CCL2/CCR2 signaling is essential to the repair of acute injury to skeletal muscle; CCL2−/− or CCR2−/− mice displayed markedly reduced muscle regeneration [3, 26]. CCL2 −/− mice demonstrated abnormalities in monocyte recruitment and cytokine expression [27]. CCR2−/− mice also exhibited a severe reduction in leukocyte adhesion and monocyte extravasation [28]. Muscle regeneration and adipocyte accumulation were significantly impaired in CCL2−/− mice compared with wild-type mice; however, muscle regeneration and adipocyte accumulation impairments were not as severe as observed in CCR2−/− mice [7]. Moreover, as mentioned earlier, Haiyan Lu et al. [14] revealed that the muscle volume of CCL2−/− mice decreased, as did the recovery area of damaged muscle. As evidenced by many studies, recruitment of macrophages to injury sites is crucial for skeletal muscle regeneration [26, 29, 30]. It has also been reported that CCL2 single nucleotide polymorphisms (SNPs) and CCR2 SNPs are related to skeletal muscle volume, strength, and changes in muscle volume and strength following resistance training [16]. In addition, a previous study using the heterochronic parabiosis model (connecting young and old mice to confirm aging-related factors) reported that CCL2 increased in both old mice and the parabiosis model [5]. Those previous studies are consistent with our findings. Taken together, the evidence suggests that an increase in CCL2 in the context of aging or muscle damage is compensatory.

We confirmed that CCR2 mediates CCL2, and when CCR2 was inhibited, the effect of CCL2 in promoting muscle differentiation was suppressed. CCR2, also called CD192 (cluster of differentiation 192), is a protein encoded by the *CCR2* gene in humans [31]. *CCR2* is located in the chemokine receptor gene cluster region and encodes two isoforms of a receptor for monocyte chemoattractant protein-1 (MCP-1, CCL2), a chemokine that mediates monocyte chemotaxis [32]. Because the *CCR2* gene encodes two isoforms of the receptor, CCR2 inhibition could produce a combination of properties. Therefore, the results that partly conflict with our findings could be due to the confounding effect of CCR2 inhibition.

There have been differing reports about the effects of CCR2 inhibition on the recovery of damaged muscles. According to one report, CCR2 inhibition has transient benefits by modulating inflammatory cell populations in chronic muscle degenerative disorder [33–35]. In addition, Roméo S. Blanc et al. showed that a high level of CCR2 ligand during adult muscle regeneration is consistent with recruitment of inflammatory cells and satellite cell expansion [36]. Furthermore, the later stages of adult muscle regeneration, including resolution of inflammation, lead to muscle differentiation, which has been associated with decreased CCR2 ligand levels [36]. At first glance, this may seem contradictory to our findings. However, because CCR2 inhibition at the late stage after injury enhances aging regeneration and functional recovery, timely CCR2 inhibition could promote recovery in aging muscles. Thus, the role of the CCR2 ligand differs depending on the time of action after muscle injury [37]. In this study, we confirmed the effect of CCL2 on myogenesis and its action through its receptor, CCR2. Moreover, muscle damage induced by dexamethasone was restored by CCL2 treatment. In addition, we confirmed that CCL2 mediated the stimulation of AKT-mTOR signaling in myogenesis. The specific mechanism of age-related systemic CCL2 elevation is not yet understood, and it would require further investigation.

Because skeletal muscle injury causes the accumulation of cell types other than muscle stem cells, the cell types and numbers identified in resting skeletal muscle differ from those identified at different stages of regeneration [38–40]. During muscle regeneration, the macrophage phenotype was switched from pro-inflammatory to anti-inflammatory macrophages [26, 41], and fibroadipogenic progenitor cells (FAPs) emerged as key regulators of skeletal muscle regeneration [42, 43]. FAPs aid the differentiation of satellite cells (SCs) in a paracrine manner during tissue regeneration [43] and are known to interact with other cells in the muscle stem cell niche to repair muscle [39, 42, 44]. As such, it has been revealed through recent studies that fibrotic differentiation of FAPs is a significant factor in muscle cell differentiation and regenerative myogenesis [45]. And various paracrine factors are related to the activity of FAPS. Therefore, it is also an important and interesting topic to determine whether CCL2 mentioned in this paper may affect the activity of FAPS. Further research on this topic is needed in the future.

In this study, with the effects of glucocorticoid-induced skeletal muscle atrophy in mind, we conducted an experiment to determine whether CCL2 restored muscle atrophy due to dexamethasone administration. However, although it is rare, we must consider that one study found that dexamethasone treatment in MBs promoted C2C12 MTs differentiation [46]. For reference, in this study, dexamethasone also induced MTs atrophy after differentiation and at the MTs stage. However, administration of dexamethasone at the onset of differentiation was found to enhance the process of MTs differentiation. C2C12 cells shift from MBs to MTs through several steps and there are many factors involved in this process. Therefore, it should be kept in mind that the effects of the same drug may vary depending on the timing of administration.

The advantage of this study is that it revealed that CCL2 promotes myogenesis and that the CCR2-CCL2 system is a crucial pathway. In addition, the positive role of CCL2 in myogenesis was verified because CCL2 restored damaged muscles. We also confirmed that the AKT-mTOR signal pathway mediates myogenesis in this process. Nonetheless, some limitations should be considered when interpreting our data. First, since we only performed experiments on the C2C12 and HSMM cell line, further experiments on primary human myoblasts isolated from young or elder age groups are needed. Second, confirmation of the effect of dexamethasone on MBs as well as MTs would be helpful for understanding the mechanism of CCL2 in myogenesis as a whole. Third, it is also necessary to study the effects of another cytokine family that may be related and cells of other lineages, such as FAPs, which are involved in the muscle injury process. Fourth, because this was an *in vitro* study, it is necessary to confirm the relationship between CCL2 and muscle mass or strength under *in vivo* conditions. Fifth and most importantly, despite the positive effect of CCL2 on MBs and MTs in our *in vitro* results, which suggest that CCL2 is a potential therapeutic target for low muscle mass and/or sarcopenia, the relationship between CCL2 or CCR2 expression and sarcopenia parameters in humans remains to be determined. Further studies are needed to confirm these relationships.

In conclusion, CCL2 has positive effects on myogenesis through CCR2. We confirmed that CCL2 has potential in the prevention and reversal of muscle mass loss through compensatory increases related to aging. Further experiments are needed to clarify the exact mechanisms by which CCL2 plays a beneficial role in skeletal muscle cells.

## MATERIALS AND METHODS

### Cell culture and reagents

Mouse C2C12 cells were maintained in growth medium (DMEM, Welgene, Gyeongsan-si, Korea) containing 10% fetal bovine serum (FBS; Gibco-BRL, Waltham, MA, USA), 1% v/v penicillin–streptomycin (10,000 U/mL of penicillin and 10,000 g/mL of streptomycin, Gibco-BRL) at 37° C in a humidified atmosphere containing 5% CO2. Differentiation was induced by switching to a low serum medium (DMEM + 2% horse serum (Gibco-BRL), 1% v/v penicillin–streptomycin). The medium was replaced every day.

HSMM (Lonza, CC-2580, Walkersville, MD, USA) were cultured in skeletal muscle growth media-2 (Lonza, CC-3246) supplemented with SingleQuots™ Kit (human epidermal growth factor, Dexamethasone, L-glutamine, FBS, and Gentamicin/ Amphotericin-B; Lonza, CC-3244) and incubated under 5% CO2 at 37° C. To study MTs, after 1–2 days (when the cells reach 80–90% of confluence) of seeding with growth media, the cells were cultured in a differentiation media consisting of DMEM: F-12 (Lonza, 12-719F, Walkersville, MD, USA) supplemented with 2% horse serum. During the complete differentiation process (5 days), cells were exposed to hCCL2 (R&D Systems, Minneapolis, MN, USA) or vehicle control (0.1% BSA in PBS) and incubated in the same differentiation media. The differentiation media was changed every 48 hours with hCCL2 or vehicle control. For the dexamethasone test, after MTs were fully differentiated for 3-4 days, the cells were cotreated with CCL2 or hCCL2 (100, 200 ng/ml) and dexamethasone 200 μm for 24 h. Recombinant mouse CCL2/JE/MCP-1 and hCCL2 were purchased from R&D Systems (Minneapolis, MN, USA). Dexamethasone was purchased from Sigma Aldrich (St. Louis, MO, USA).

### Cell viability assay

Cell viability was measured using a CCK-8 assay (Dojindo, Kumamoto, Japan) according to the manufacturer’s instructions. MBs cells (1×10^4^ cells/well, 100 μl) were seeded into 96-well plates for 24 h. Fresh serum free medium containing the indicated concentrations of CCL2 or hCCL2 was added. The control ‘0’ was treated with vehicle of CCL2 or hCCL2 only (0.1% BSA in PBS). After incubation for 24 h, culture medium was replaced with drug-free medium (100 μl). Briefly, 10 μl of WST-8 dye (2-[2-methoxy-4-nitrophenyl]-3-[4-nitrophenyl]-5-[2, 4-disulfophenyl]-2H-tetrazolium, monosodium salt) was added to each well of a 96-well plate [47]. The suspension was incubated for 2 h, and the absorbance was read at 450 nm, with a reference wavelength of 650 nm, using a microplate reader (BioTek Epoch Gen5, Houston, TX, USA).

### Proliferation assay

C2C12 MBs (1×10^4^ cells/well, 100μl) were seeded into 96-well plates for 24 hours. Fresh serum free medium containing the indicated concentrations of CCL2 was added. Cell proliferation was measured by monitoring BrdU incorporation. Cells were incubated with BrdU for 3 hours, and then cell proliferation was assayed using a BrdU labeling and detection kit (Roche, Mannheim, Germany). Absorbance was measured at 450 nm, with a reference wavelength of 650 nm, using a microplate reader (BioTek Epoch Gen5).

### Migration assay

To examine cell migration capacity, we used a Boyden chamber system with Transwell plates and 8-μm-porosity polycarbonate membranes (Corning, NY, USA). Cells were seeded onto the inner chamber at a density of 1.0 × 10^5^ cells/200 μL in DMEM with 0.1% FBS and then exposed to CCL2 (100 and 200 ng/ml) in the outer chamber for 24 hours [48]. Cells on the upper membrane were removed by wiping with a cotton swab, whereas cells on the lower surface of the membrane were fixed in 4% paraformaldehyde (PFA) for 2 min and stained with 0.05% crystal violet. Images of the lower side of the membrane were produced using a microscope (Olympus CKX-41, Tokyo, Japan). The total number of migrated cells for each well was quantified using ImageJ software.

### Quantitative reverse-transcription PCR (qRT-PCR)

Total RNA was extracted from C2C12 or HSMM cells with TRIzol reagent (Sigma Aldrich, St. Louis, MO, USA). We used RNA to cDNA EcoDry Premix (Takara, Shiga, Japan) to carry out cDNA synthesis for mRNA. The analysis of mRNA expression was carried out with Faststart Essential DNA Green Master (Roche, Mannheim, Germany) in a Roche Light Cycler 96 system (Roche, Mannheim, Germany) following the manufacturers’ instructions. Primers for anti-MyHC (NM_001039545.2, BC093082.1), myogenin (NM_031189.2, NM_002479.6), MyoD (NM_010866.2), Myf5 (NM_008656.5), MRF4 (NM_008657.2), PCNA (NM_011045.2), Cyclin D1 (BC044841.1), and CCR2 (NM_009915.2) were obtained from Applied Biosystems (Foster City, CA, USA). The threshold cycle (Ct) value for each gene was normalized to the Ct value of glyceraldehyde 3-phosphate dehydrogenase (GAPDH) (M_008084.3, BC083511.1).

### Western blot analysis

C2C12 cells were lysed at 4° C in RIPA lysis buffer (Enzo Life Sciences, Farmingdale, NY, USA), and the lysate was centrifuged at 13,000 rpm and 4° C for 20 min. Aliquots of the supernatant were removed for protein analysis by the Bicinchoninic acid (BCA) protein assay method (Pierce, Rockford, IL, USA). The supernatant was incubated at 95° C for 5 min in 4x Laemmli Sample Buffer (Bio-Rad, Laboratories, Hercules, CA, USA) containing 355mM 2-mercaptoethanol, and 20μg of the sample was resolved by 10% SDS-PAGE. The separated proteins were transferred to a polyvinylidene fluoride (PVDF) membrane (Merck Millipore, Molsheim, France). The primary antibodies [phospho-mTOR (Ser2448), mTOR, phospho-Akt (Ser473), and Akt] and HRP-conjugated secondary antibodies (HRP-linked anti-mouse IgG and anti-rabbit IgG) were purchased from Cell Signaling Technology (Danvers, MA, USA). Primary antibodies against anti-MyHC and anti-α-tubulin were purchased from Sigma Aldrich. Myogenin and CCR2 primary antibodies were purchased from Abcam (Cambridge, UK). Immunoreactive bands were visualized using a LAS-AI600 enhanced chemiluminescence detection system (Amersham Pharmacia Biotech, Arlington Heights, IL, USA). Protein expression levels were normalized to that of α-tubulin, and the western blotting band signal intensities were quantified using ImageJ software.

### Immunofluorescence

C2C12 or HSMM cells were grown on coverslips (SPL Life sciences, Phocheon-si, Korea, Cat#20018). To evaluate the protective effects of CCL2 or hCCL2 on dexamethasone-induced muscle atrophy, differentiated MTs were cotreated with recombinant CCL2 or hCCL2 (100, 200 ng/ml) and dexamethasone (200 μm) for 24 hours. C2C12 or HSMM MBs and MTs were fixed in ice-cold 4% PFA for 15 min, washed three times with PBS, and incubated in ice-cold 0.25% Triton X-100 at room temperature for 10 min [49]. Then, cells were blocked with blocking solution (1% BSA in PBS) and washed three more times. Next, cells were incubated overnight in anti-MyHC (1:400, Sigma Aldrich, St. Louis, MO, USA) at 4° C. Following three washes in PBS, the cells were incubated with Alexa 555–labeled anti-mouse IgG antibodies (1:1000, Cell Signaling, MA, USA) for 60 min. Then, cells were stained with 4, 6-diamidino-2-phenyindole (DAPI, 1:5000, Sigma Aldrich, St. Louis, MO, USA) for 10 min and washed with PBS three times. The images were produced using an LMS800 fluorescence microscope (Carl Zeiss, Oberkochen, Germany). The fusion index (%) was calculated using the following equation: 100×number of nuclei in MyHC+ MTs per total number of nuclei in MyHC+ myocytes and MTs.

### siRNA transfection

C2C12 MB cells (1.5×10^5^ cells/well) were seeded into 6-well plates for 24 h. CCR2 siRNA oligonucleotides (NM_009915.2), CCR4 siRNA oligonucleotides (NM_009916.2), and negative control siRNA oligonucleotides were synthesized by Bioneer Corp. AccuTarget™ negative control siRNA (SN-1001) was also purchased from Bioneer (Daejeon, Korea). siRNA oligonucleotides were transfected into C2C12 cells using Lipofectamine 3000 (Invitrogen, Waltham, MA, USA) as advised by the manufacturer’s protocol. The siRNA concentration used was 100nM. Briefly, each siRNA and lipofectamine 3000 (Invitrogen) was diluted in serum-free OPTI-MEM and incubated for 5 min, respectively. The diluted siRNA and lipofectamine 3000 reagent were mixed by inverting and incubated for 20 min to form complexes. Pre-formed complexes were added directly to the cells and cells were incubated for an additional 6 h. Then, culture medium was replaced with antibiotic and 10% FBS-containing DMEM and incubated. The next day, differentiation media and CCL2 200 ng/ml were administered together. We used MTs from day 3 of differentiation for our experiments.

### MK2206-mediated Akt inhibition

C2C12 cells were treated with 5 μM specific Akt inhibitor MK2206 (MedChemExpress, HY-10358, Monmouth Junction, NJ, USA) and 200 ng/ml CCL2 for three days of differentiation. Re-adding MK2206, CCL2, or vehicle control (DMSO) changed daily.

### SUnSET assay by Western blotting

To measure *in vitro* protein synthesis using the SUnSET assay, C2C12 cells were treated with 1 μM puromycin (P8833, Sigma Aldrich) for 30 min before harvesting. Cell lysates were normalized for equal amounts of protein using the BCA Protein assay method (Pierce). Samples were boiled with 4x Laemmli Sample Buffer (Bio-Rad) containing 355 mM 2-mercaptoethanol, and 10 μg of the sample was resolved by 10% SDS-PAGE. The proteins were transferred onto a 0.45 μm PVDF membrane (Merck Millipore). Blocking was conducted for 30 min with 3% skim milk prepared in Tris-buffered saline with 0.05% Tween 20 (TBST). Blots were washed thrice in TBST and incubated with anti-puromycin (1:25000, Millipore, MA, USA) as the primary antibody. Membranes were washed thrice with TBST and incubated with HRP-conjugated anti-mouse IgG (Cell Signaling Technology, Danvers, MA, USA) as the secondary antibody. Immunoreactive bands were visualized using a LAS-AI600 enhanced chemiluminescence detection system (Amersham Pharmacia Biotech, Arlington Heights, IL, USA). Protein expression levels were normalized to that of α-tubulin, and western blotting band signal intensities were quantified using ImageJ software.

### Statistical analysis

The data are expressed as mean ± standard deviation (SD) from at least three independent experiments relying on triplicate measurements, unless otherwise specified. We checked the normality using the Shapiro-Wilk test. For items that satisfied normality, Student’s t test and analysis of variance (ANOVA) with post hoc analysis via Tukey’s honest significance test were used to evaluate the significance of differences between two groups and among ≥3 groups, respectively. For items that did not satisfy normality, the Mann-Whitney U test and the Kruskal Wallis test were used to evaluate the significance of differences between two groups and among ≥3 groups, respectively. P values <0.05 were considered statistically significant. SPSS version 20.0 (SPSS, Inc., Chicago, IL, USA) was used for statistical analyses.

## Supplementary Material

Supplementary Figures
