# Supplementary material for: C-C motif chemokine ligand 2 promotes myogenesis of myoblasts via the AKT-mTOR pathway
Source: Aging (Albany NY). 2022 Dec 27;14(24):9860–76. doi: 10.18632/aging.204451 (PMC9831732; doi:10.18632/aging.204451)
Supplement: Supplementary Figures [file aging-14-204451-s001.pdf]

SUPPLEMENTARY FIGURES

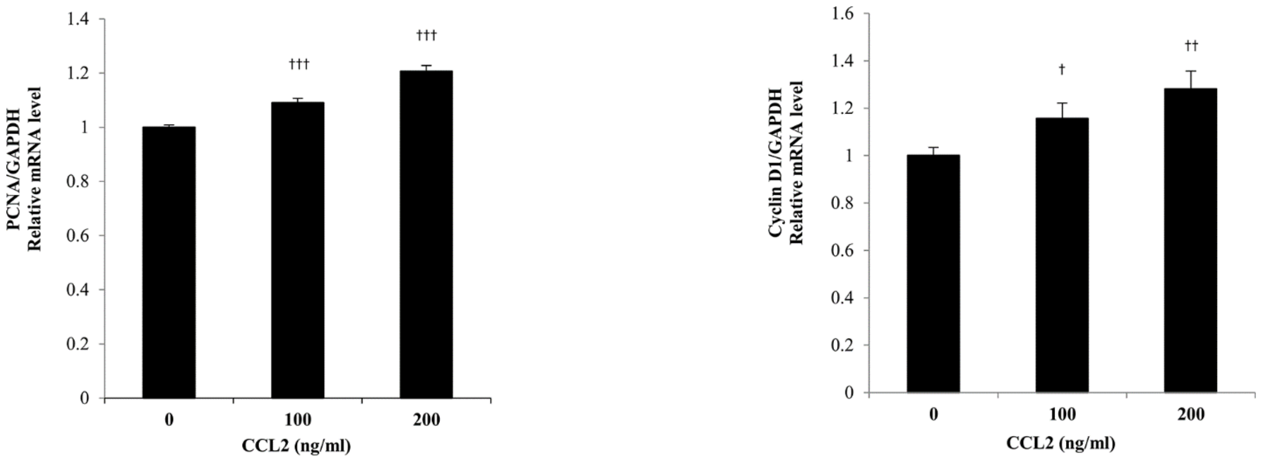

† P<0.01, †† P<0.001, ††† P<0.0001, (vs CCL2 '0')

Supplementary Figure 1. Re-confirmation of the effect of cell proliferation of C-C motif chemokine ligand 2 (CCL2) using the relative expression of PCNA and cyclin D1. Quantification of relative expression of PCNA and Cyclin D1 after treatment with 0nM, 100nM, or 200 nM CCL2. Number of experiments (No.) = 4 times. Data are expressed as mean ± standard deviation (SD). *P<0.05, **P<0.005, ***P<0.0005, †P<0.01, ††P<0.001, †††P<0.0001 vs. day 1 of CCL2 '0' (vehicle: PBS + 0.1% BSA).

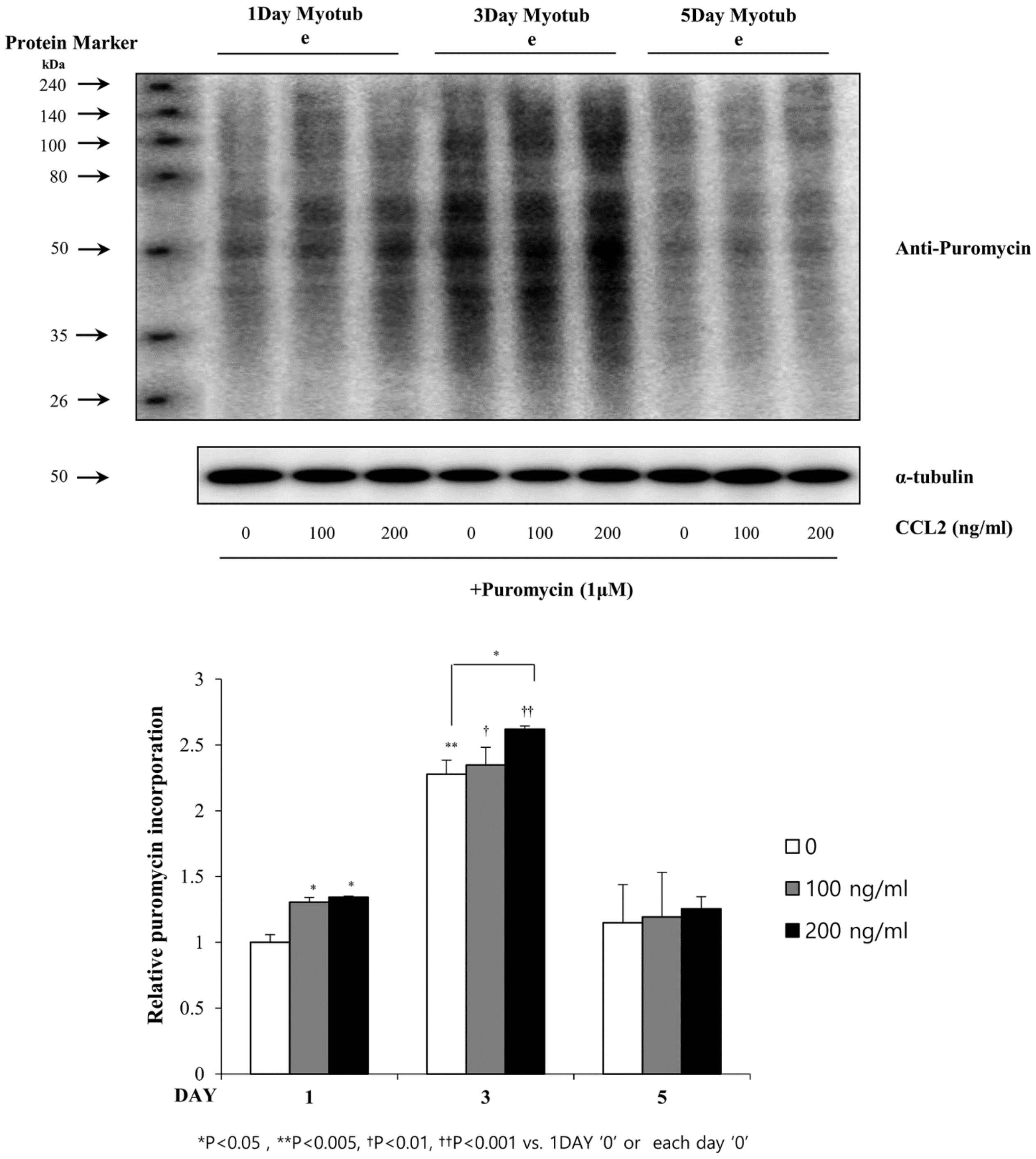

Supplementary Figure 2. The effect of C-C motif chemokine ligand 2 (CCL2) on protein synthesis during myogenesis. Quantification of relative puromycin incorporation in the SUnSET assay. Western blot analysis to determine protein synthesis activity in C2C12 cells during myogenesis after treatment with 100nM or 200 nM CCL2 per day. Number of experiments (No.) = 4 times. Data are expressed as mean ± standard deviation (SD). *P<0.05, **P<0.005, ***P<0.0005, †P<0.01, ††P<0.001, †††P<0.0001 vs. day 1 of CCL2 '0' (vehicle: PBS + 0.1% BSA), or vs. same day data (represented with lines).

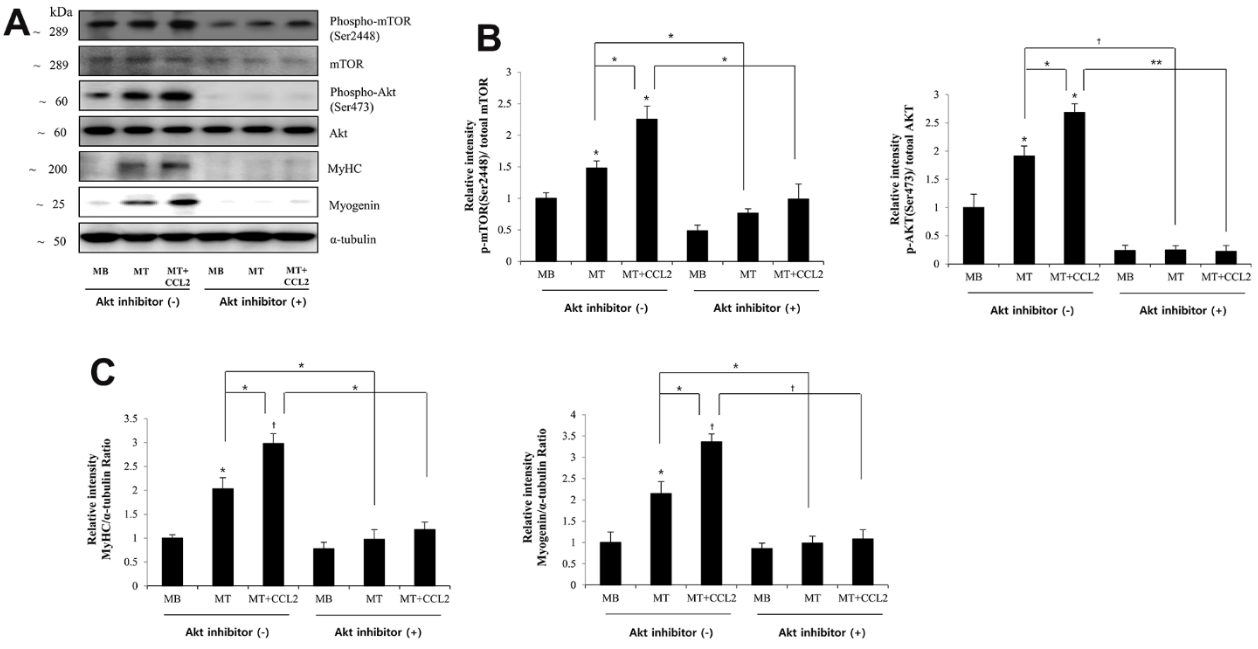

* P<0.05, ** P<0.005, † P<0.01 (vs without MB or without MT, or without MT&CCL2)

Supplementary Figure 3. Effects of C-C motif chemokine ligand 2 (CCL2) on myogenesis are mediated by AKT-mTOR signal stimulation, proven with Akt inhibitor. (**A**) Western blot analyses band to determine the activity of signals related to myogenesis in C2C12 cells after treatment with 200 ng/ml CCL2 with Akt inhibitor. (**B**) Western blot analyses of the relative intensity of *p*-mTOR/mTOR or *p*-AKT/total AKT in C2C12 cells treated with 2% horse serum in the presence or absence of CCL2 and/or Akt inhibitor for three days. (**C**) Western blot analyses of the relative intensity of myosin heavy chain (MyHC) or Myogenin in C2C12 cells treated with 2% horse serum in the presence or absence of CCL2 and/or Akt inhibitor for three days. Number of experiments (No.) of (**A**–**C**) = 4 times, respectively. Data are expressed as mean ± standard deviation (SD). (**B**, **C**) *P<0.05, **P<0.005, †P<0.01, ††P<0.001 vs. Akt inhibitor '–' MB, or vs. Akt inhibitor '–' MT, or Akt inhibitor '–' MT & CCL2 (represented with lines).
